# Early development of physical co-morbidity and premature death in long-term first-episode psychosis cohorts

**DOI:** 10.1192/j.eurpsy.2025.131

**Published:** 2025-08-26

**Authors:** J. Vázquez-Bourgon, PAFIP & ITPCan

**Affiliations:** Marques de Valdecilla University Hospital, Santander, Spain

## Abstract

**Abstract:**

People with psychosis present a significantly higher premature death, leading to a reduction of life expectancy of up to 15-20 years.

To a high extent, this premature mortality is due to a higher incidence of common physical conditions, appearing earlier than in the general population. Traditionally, the focus has been put on the cardiovascular diseases. However, more recently, there ir mounting evidence of the contribution of other conditions to this premature mortality, such as repiratory (e.g.: chronic obstructive pulmonary disease) and liver (MAFLD) conditions.

We are presenting results from PAFIP and ITPCan early intervention programs (Cantabria, Spain), regarding the early impact of psychosis on physical health, observing early alterations at the organic level (e.g.: liver, lung), which precede the development of chronic organic pathology causing premature mortality in the general population.

**Disclosure of Interest:**

None Declared

